# Quantifying ocular microcirculation in hypertension patients with carotid artery stenosis

**DOI:** 10.3389/fnins.2024.1361413

**Published:** 2024-07-22

**Authors:** Jinlan Ma, NanJia Gelie, Mingjuan Zhu, Xiaolu Ma, Changjing Han

**Affiliations:** ^1^Department of Ophthalmology, Affiliated Hospital of Qinghai University, Xining, China; ^2^Department of Ultrasound, Affiliated Hospital of Qinghai University, Xining, China; ^3^Department of Ophthalmology, Traditional Chinese Medicine Hospital of Qinghai Province, Xining, China; ^4^Department of Ophthalmology, Haidong First People’s Hospital, Pingan, China

**Keywords:** hypertension, carotid artery stenosis, vessel density, choroidal vascularity index, central retinal vein equivalent

## Abstract

**Background:**

Carotid artery stenosis (CAS) is one of the most common macrovascular complications of hypertension. The ophthalmic artery springs from the internal carotid artery; however, the effect of CAS on ocular microcirculation has not been quantified in hypertension patients. This study aimed to quantify ocular microcirculation metrics in hypertension with CAS (HCAS) patients and to explore the relationship between micro- and macroangiopathy in hypertension.

**Methods:**

All participants (community-based) underwent detailed assessments, including carotid ultrasonography, optical coherence tomography angiography (OCTA), and enhanced depth imaging (EDI)-OCT. CAS was diagnosed using carotid ultrasonography. Retinal microcirculation metrics, including vessel density (VD), skeleton density (SD), fractal dimension (FD), and foveal avascular zone (FAZ), were quantified using OCTA and ImageJ software. Choroidal microcirculation metrics, including subfoveal choroidal thickness (SFCT), luminal area (LA), and choroidal vascularity index (CVI), were quantified using EDI-OCT and ImageJ. Retinal vessel caliber metrics, including central retinal artery equivalent (CRAE), central retinal vein equivalent (CRVE), and artery/vein ratio (AVR), were calculated using revised formulas. The above metrics were compared among the HCAS group, hypertension with no CAS (HNCAS) group, and healthy control group. The mutual effects between ocular metrics and CAS were evaluated using regression analyses.

**Results:**

In a comparison of the HCAS vs. HNCAS groups, retinal metrics including VD, SD, FD, and choroidal metrics including CVI and LA were significantly decreased in the HCAS group (all *p* < 0.05); however, FAZ, SFCT, and retinal vessel caliber metrics including CRAE, CRVE, and AVR were comparable between groups (all *p* > 0.05). In a comparison of HNCAS and the healthy control group, VD, SD, and CRAE showed that AVR was significantly decreased in the HNCAS group (all *p* < 0.05); meanwhile, choroidal metrics were comparable between groups (all *p* > 0.05). Linear regression analyses showed that intima-media thickness (IMT) (*p* = 0.01) and peak systolic velocity (PSV) (*p* = 0.002) were negatively related to retinal VD in hypertension patients. Logistic regression analyses disclosed that older age (*p* < 0.001), smoking history (*p* = 0.002), lower VD (*p* = 0.04), SD (*p* = 0.02), and CVI (*p* < 0.001) were related to the presence of CAS in hypertension patients.

**Conclusion:**

CAS in hypertension-induced hypoperfusion in retinal and choroidal microcirculation and the decreased retinal VD and choroidal CVI were significantly associated with the presence of CAS in patients with hypertension, suggesting that hypertension macro- and microangiopathy were mutually affected and share the common pathophysiology. Furthermore, OCT could be a useful tool to assess hypertension patient’s CAS risk profiles in a non-invasive way.

## Introduction

Hypertension could induce macrovascular and microvascular complications; therefore, screening for vascular complications ahead of irreversible damage in high-risk patients is important to save the individual and public financial expenses on hypertension (Laurent et al., 2022). Carotid artery stenosis (CAS) is one of the most common macrovascular complications of hypertension, which has been reported to be responsible for approximately 10–20% of ischemic strokes (Buus et al., 2013). Over the past few decades, lots of efforts have been made to develop objective, non-invasive, and earlier biomarkers for CAS. Ocular microcirculation metrics have been reported as indicators of microvascular complications in hypertension because they provide a unique window to observe microangiopathy non-invasively and objectively (Xu et al., 2021). Traditionally, macro- and microvascular complications of hypertension were considered unrelated and distinct. Recently, pathophysiological and epidemiologic evidence suggested that macro- and microvascular complications of hypertension may be correlated and mutually affected (Krentz et al., 2007).

The development of optical coherence tomography angiography (OCTA) has allowed for the acquisition of retinal and choroidal microcirculation objectively and non-invasively (You et al., 2017). More recently, different retinal microcirculation metrics, including vessel density (VD), skeleton density (SD), foveal avascular zone (FAZ), and fractal dimension (FD), were extracted from OCTA images as biomarkers for evaluating retinal vasculopathy in diabetes (Samara et al., 2017), hypertension (Xu et al., 2021), high-altitude retinopathy (Ma et al., 2022), and uveitis (Kim et al., 2016). Meanwhile, benefiting from the enhanced depth imaging (EDI)-OCT technique, we can measure subfoveal choroidal thickness (SFCT) and calculate the choroidal vascularity index (CVI) as effective biomarkers for evaluating the choroidal vasculopathy in pachychoroid diseases (Agrawal et al., 2016a), age-related macular degeneration (Koh et al., 2017), and high-altitude retinopathy (Ma et al., 2022). Parr and Spears (1974) suggested that blood flow is proportional to the lumen of vessels rather than caliber, and the number of branches could influence the lumen of vessels; hence, the central retinal artery equivalent (CRAE) and central retinal vein equivalent (CRVE) were summarized to quantify the lumen of central retinal vessels from fundus photography. Then, Hubbard et al. (1999) raised a revised formula to calculate CRAE and CRVE, which was more accurate and reliable. Recently, studies verified that CRVE and CRAE were linearly correlated with blood pressure (BP) in hypertension (Köchli et al., 2019; Meyer et al., 2020).

The ophthalmic artery springs from the internal carotid artery and supplies blood to the retina and choroid. Hence, carotid disease may exert an influence on the blood perfusion of the retina and choroid. Several studies have demonstrated that carotid parameters, including intima-media thickness (IMT) and peak systolic velocity (PSV), were significantly related to retinal microvascular metrics in CAS (Xu et al., 2022) and diabetes (Drinkwater et al., 2020); however, data on the association between carotid ultrasonographic parameters and ocular metrics, including retinal microcirculation metrics, choroidal microcirculation metrics, and retinal vessel caliber metrics, in hypertension patients had never been reported.

Thus, this study aimed to compare the retinal microcirculation metrics, choroidal microcirculation metrics, and central retinal vessel caliber metrics among hypertension with CAS (HCAS) patients, hypertension with no CAS (HNCAS) patients, and a healthy control group, so as to elucidate the mutual effect between macro- and microangiopathy in hypertension and to identify risk factors and useful biomarkers for the presence of CAS in hypertension patients.

## Methods

### Study population

This community-based, prospective observational study was carried out in the Guchengtai community, Xining City, Qinghai Province, China, from January 2022 to December 2023. Patients with primary hypertension and healthy subjects over the age of 50 years from the Guchengtai community were included in the study after giving informed consent. Approval was obtained from the Ethics Committee of Qinghai University; in addition, the study adhered to the tenets of the Declaration of Helsinki.

### Clinical assessment

All participants underwent detailed assessment, including medical history, physical examination, color fundus photography, OCT and OCTA, and carotid ultrasonography, conducted in a single visit. BP was measured by two well-trained nurses using a standardized mercury sphygmomanometer. The mean arterial pressure (MAP) and mean ocular perfusion pressure (MOPP) were calculated as follows: MAP = Diastolic BP+[1/3 (systolic BP-diastolic BP)] (McCorry, 2007), MOPP = 2/3 × (MAP-IOP) (Baek et al., 2023).

All participants underwent dilated ophthalmic examination, including best corrected visual acuity (BCVA) recorded in the logarithm of the minimum angle of resolution (log MAR), refraction, slit lamp examination, intraocular pressure (IOP) measurement, and fundus photography before obtaining OCTA/EDI-OCT scans.

The inclusion criteria for HCAS are as follows: (1) Primary hypertension patients whose BP is between 140/90 mmHg and 180/110 mmHg from triplicate office measurement, no history of cardiovascular diseases, diabetes mellitus, renal insufficiency, or other concomitant disease; (2) Unilateral asymptomatic CAS diagnosed according to the criteria provided by the Society of Radiologists in Ultrasound Consensus Conference (Grant et al., 2003); and (3) Spherical equivalent (SE) not exceeding ±3.0 diopters, IOP not exceeding 21 mmHg, and BCVA not lower than 0.3.

The inclusion criteria for HNCAS are as follows:1. Primary hypertension patients without concomitant disease who are between the ages of 50 and 70 years; 2. There is no plaque or intimal thickening in carotid ultrasonography, and the PSV is less than 125 cm/s; and 3. They have a healthy ocular status.

Healthy control subjects were between 50 and 70 years old and had normal ocular status, without hypertension, CAS, diabetes mellitus, or any other diseases.

Participants with secondary hypertension, malignant hypertension, and bilateral CAS were excluded. Individuals with cloudy refractive structures and concomitant retinal diseases were also excluded. Finally, the right eye in the control group or the ipsilateral eye of CAS in the HCAS and HNCAS groups were included for further analyses.

### OCTA image acquisition and processing

**Figure 1:**
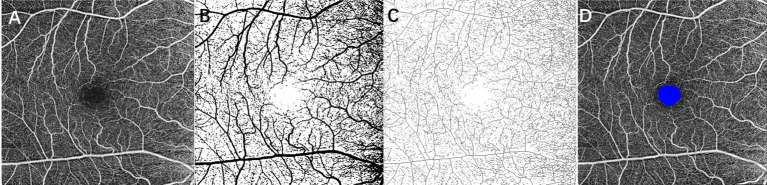
Extracting retinal microcirculation metrics from 6 × 6 mm OCTA image at the superficial layer of the retina. **(A)** Original grayscale image; **(B)** binarized image for measurement of vessel density; **(C)** skeletonized image for calculating skeleton density; and **(D)** foveal avascular zone (FAZ) was outlined as blue.

OCTA images of a 6 × 6 mm macular area were acquired using Zeiss Cirrus HD-OCT (Carl Zeiss Meditec, Inc., United States). Based on automated software default settings, the superficial retinal layer (SRL) extended from 3 μm below the internal limiting membrane to 15 μm below the inner plexiform layer. In the current study, we only processed the SRL images because shadow artifacts from SRL on the deep capillary plexus cannot be removed completely at 6 × 6 mm views and may misguide the results. The scans centered on the fovea with signal intensity ≥7 were included for analysis. Retinal microcirculation metrics, including VD, SD, FD, and FAZ, were obtained using ImageJ software (National Institutes of Health [NIH], Bethesda, MD, United States), as shown in Figure 1 (Zahid et al., 2016; Ma et al., 2022). VD was defined as the VD in a macular 6 × 6 mm view and calculated as the percentage of area occupied by blood vessels in the entire en-face scan. To allow the quantification of VD, the obtained OCTA scans were binarized using an auto-thresholding algorithm, with the blood vessels being defined as pixels having decorrelation values above the threshold level (Figure 1B). Then skeletonizing the binarized scan to generate 1-pixel wide vascular tree and to provide an indication of the SD independent of vascular diameter (Figure 1D). SD was then calculated as a percentage of the total vascular length divided by the total area. The FAZ was a sign of foveal ischemia. The FAZ was manually outlined in ImageJ and calculated using the ROI plugin (Figure 1D). FD was defined as the FD of the OCTA image and calculated as an indicator of the branching complexity of small vessels using the box-counting method by the Fractal Box Count plugin in ImageJ. The value of FD was between 1 and 2, and a higher value of FD represents more branches of capillary (Zahid et al., 2016).

### EDI-OCT image acquisition and processing

**Figure 2:**
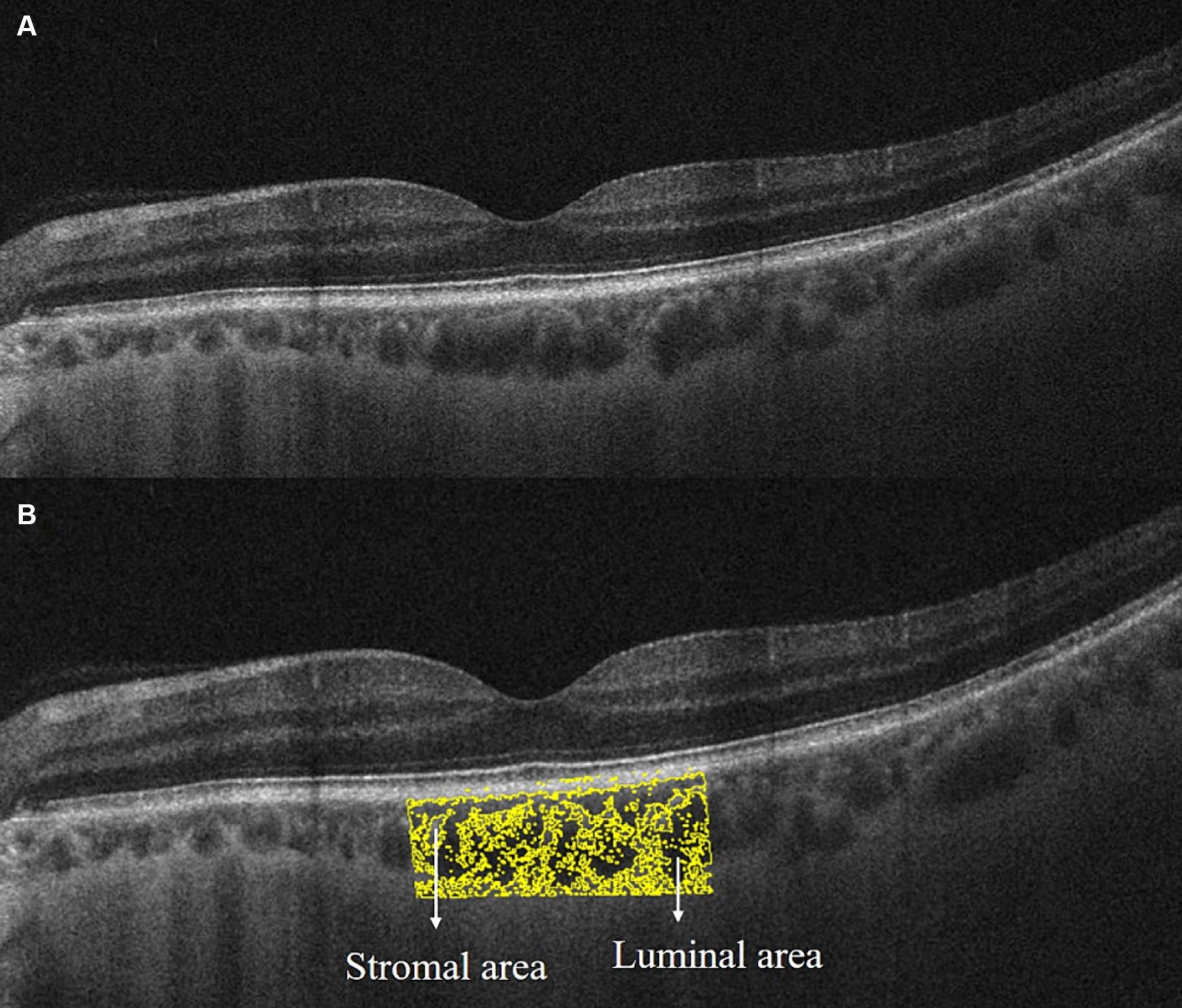
Extracting choroidal microcirculation metrics from EDI-OCT image. **(A)** Original grayscale image; **(B)** binarized image for distinguishment of luminal area (yellow pixel and dark area) and stromal area (light area) by Niblack autolocal threshold in ImageJ software.

EDI-OCT images were acquired using the built-in EDI program in OCT (Carl Zeiss Meditec, Inc., United States). Scans with signal intensity ≥ 7 and clear choroid–scleral interface were collected for analysis. The SFCT was measured from the retinal pigment epithelium to the choroid–scleral interface at the subfoveal level using a built-in caliper. Choroidal microcirculation metrics, including total choroidal area (TCA), luminal area (LA), and stromal area (SA), were quantified using ImageJ software. First, use the Niblack autolocal threshold to binarize the image to get a clear view of the choroid–scleral interface. Second, select TCA under the fovea with a width of 2 mm and add it to the ROI manager. Third, mark LA (dark area and yellow pixels) and SA (light area) by adjusting the color threshold (Figure 2). LA represented the area of vascularity and SA represented the area of non-vascularity (Agrawal et al., 2016a,b). Finally, CVI was calculated as the percentage of LA to the TCA for evaluating the vascular structure in the choroid.

### Retinal vessel caliber measurement

**Figure 3:**
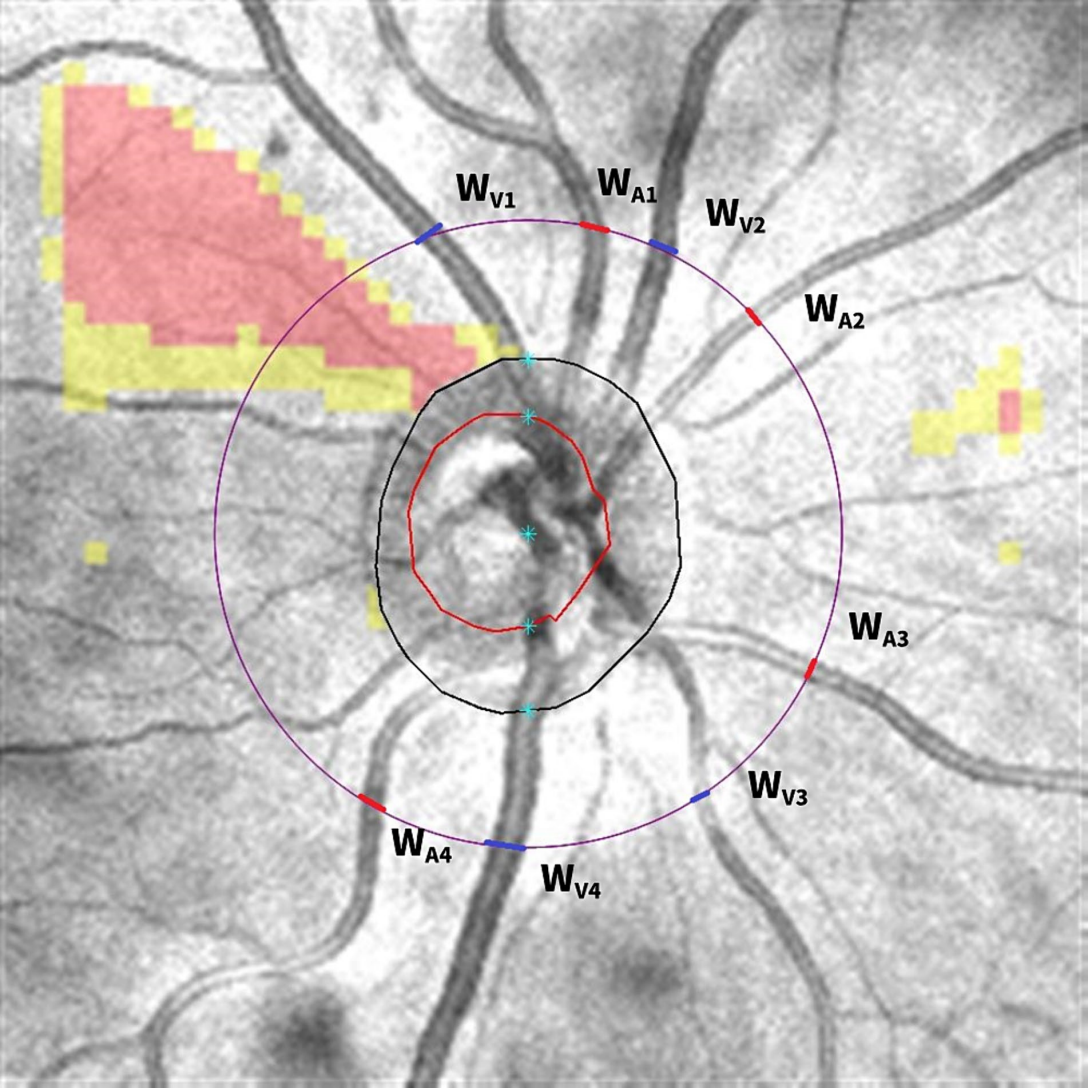
Calculating central retinal vessel equivalent from optic disk cube 200 × 200 scan. The black circle represents the area of the optic disk and the red circle represents the area of the optic cup. The diameter of the largest veins and arteries in four quadrants was measured on the purple circle, which centered on the optic disk with a diameter of 3.4 mm. The veins were marked as blue and arteries were marked as red. Then, the diameter of W_V1_/W_A1_, W_V2_/W_A2_, W_V3_/W_A3_, and W_V4_/W_A4_ was put into the equation to calculate the central retinal vein equivalent and central retinal artery equivalent.

Optic disk cube 200 × 200 scan was acquired by OCT to calculate the central retinal vessel equivalent (Figure 3). The black circle represents the area of the optic disk and the red circle represents the area of the optic cup. The largest veins and arteries in four quadrants were marked as W_V1-4_/W_A1-4_, and the widths of vessels were measured by ImageJ on the purple circle, which centered on an optic disk with a diameter of 3.4 mm as the prior study described (Ma et al., 2023). We adopted formulas advised by Knudtson et al. (2003) to calculate the CRAE and CRVE:


$$\mathrm{CRAE}:\omega =0.88\times \sqrt{{W_{1}}^{2}+{W_{2}}^{2}}$$

$$\mathrm{CRVE}:\omega =0.95\times \sqrt{{W_{1}}^{2}+{W_{2}}^{2}}$$

ω is the trunk vessel, and *w*_1_ and *w*_2_ are the widths of branch vessels. In this study, we included the largest arteries and veins in four quadrants as branch vessels and used an iterative procedure to calculate CRAE and CRVE. The artery/vein ratio (AVR) was calculated as the ratio of CRAE to CRVE.

### Carotid ultrasonography measurements

The carotid color Doppler sonography examination was performed using an iE33 echo machine (Philips Medical Systems, Andover, MA) with a high-frequency 5–12 MHz linear array ultrasound probe. The PSV of the common carotid artery and the IMT at 1 cm proximal to the carotid artery bifurcation were measured by two experienced ultrasound doctors blind to the condition of each subject. IMT was defined as the vertical distance from the upper edge of the posterior vessel wall to the upper edge of the outer membrane. Thickening of IMT was defined as IMT ≥ 1.0 mm; plaque was defined as focal IMT ≥ 1.5 mm. Grading of CAS went according to the criteria provided by the Society of Radiologists in the Ultrasound Consensus Conference (Grant et al., 2003).

### Statistical analysis

The data were analyzed using SPSS version 23 software (SPSS; IBM, Armonk, NY, United States). The normal distributions of all data were assessed using the Kolmogorov–Smirnov test. The continuous variables were reported as mean ± standard deviation (SD), and categorical variables were shown in the form of percentages. Independent-sample t-tests were used for the comparison of continuous variables between two groups and chi-square tests for categorical variables. Univariate linear regression analysis was performed to investigate the factors associated with retinal VD in HCAS and HNCAS groups, respectively. Then, multivariate linear regression with a step-wise process was performed for variables with a *p*-value of < 0.05 in the univariate regression analysis. Moreover, logistic regression analysis was conducted to explore the factors associated with CAS in hypertension patients. A *p*-value of < 0.05 was considered statistically significant.

## Results

### Baseline characteristics

After excluding participants with bilateral CAS, ocular disease, and secondary hypertension, 245 participants were included in the current study. Among them, 43 participants were excluded due to the poor-quality OCTA and EDI-OCT images. Finally, 50 eyes of 50 HCAS patients, 71 eyes of 71 HNCAS patients, and 81 right eyes of 81 age- and sex-matched healthy subjects were included for further analysis.

The baseline characteristics are listed in Table 1. The mean age was 55.51 ± 7.06 years in the HNCAS group, 58.04 ± 10.3 years in the HCAS group, and 56.65 ± 10.16 years in the control group without significant differences among groups (all *p* > 0.5). The gender ratio showed no significant differences among groups either (all *p* > 0.5). The HCAS group had a greater portion of smoking (84 vs. 64.79%, *p* = 0.02), a lower portion of anti-hypertensive treatment (62 vs.78.87%, *p* = 0.04), and longer duration of hypertension (14.3 ± 3.8 vs.11.2 ± 4.6 years, *p* < 0.001) than the HNCAS group. Carotid ultrasonography disclosed thicker IMT (1.22 ± 0.21 vs. 0.89 ± 0.18 mm, *p* < 0.001) and higher PSV (88.57 ± 26.12 vs. 75.48 ± 19.46, *p* = 0.002) in the HCAS group than the HNCAS group, and plaque was detected in 62% of subjects in the HCAS group. BMI, systolic BP, diastolic BP, MAP, and MOPP were all higher in the HCAS group and HNCAS group when compared with the healthy control group (all *p* < 0.001).

**Table 1:** Characteristics of participants.

Characteristics	HCAS *N* = 50 (eyes)	HNCAS *N* = 71 (eyes)	Control *N* = 82 (eyes)	P_1_	P_2_	P_3_
Age (years)	58.04 ± 10.3	55.51 ± 7.06	56.65 ± 10.16	0.11	0.45	0.43
Male, *n* (%)	35 (70)	51 (72.86)	60 (73.17)	0.83	0.69	0.85
Smoker, *n* (%)	42 (84)	46 (64.79)	40 (48.78)	0.02^*^	<0.001^*^	0.04^*^
Hypertension duration (years)	14.3 ± 3.8	11.2 ± 4.6	/	<0.001^*^	/	/
Anti-hypertensive treatment, *n* (%)	31 (62)	56 (78.87)	/	0.04^*^	/	/
BMI (Kg/m^2^)	23.56 ± 2.08	24.31 ± 2.45	20.78 ± 2.13	0.07	<0.001^*^	<0.001^*^
IOP (mmHg)	14.49 ± 2.16	14.74 ± 2.00	15.02 ± 2.16	0.5	0.17	0.4
SBP (mmHg)	157.66 ± 16.17	153.94 ± 12.11	120.64 ± 12.20	0.15	<0.001^*^	<0.001^*^
DBP (mmHg)	104.5 ± 17.5	101.00 ± 10.64	81.00 ± 12.64	0.17	<0.001^*^	<0.001^*^
MAP (mmHg)	122.1 ± 10.62	118.65 ± 9.89	94 ± 10.28	0.07	<0.001^*^	<0.001^*^
MOPP (mmHg)	71.7 ± 8.56	69.3 ± 8.64	53.33 ± 9.0	0.13	<0.001^*^	<0.001^*^
IMT (mm)	1.22 ± 0.21	0.89 ± 0.18	0.82 ± 0.2	<0.001^*^	0.04^*^	0.03^*^
PSV (cm/s)	88.57 ± 26.12	75.48 ± 19.46	72.34 ± 21.36	0.002^*^	<0.001^*^	0.35
Plaque, *n* (%)	31 (62)	/	/	*/*	/	/

CAS, carotid artery stenosis; HCAS, hypertension with CAS; HNCAS, hypertension without CAS; BMI, body mass index; IOP, intraocular pressure; SBP, systolic blood pressure; DBP, diastolic blood pressure; MAP, mean arterial pressure; MOPP, mean ocular perfusion pressure; IMT, intima media thickness; PSV: peak systolic velocity; P_1_, *p*-value between HCAS group and HNCAS group; P_2_, *p*-value between HCAS group and healthy controls; P_3_, *p*-value between HNCAS group and healthy controls; * Statistically significant.

### Retinal microcirculation metrics

The results of retinal microcirculation metrics are summarized in Table 2. In comparison between the HCAS group and the HNCAS group, VD, SD, and FD were significantly decreased in the HCAS group (P_VD_ = 0.02, P_SD_ < 0.001, P_FD_ < 0.001). Meanwhile, VD, SD, and FD were also significantly lower in the HCAS group when compared with the healthy group (all *p* < 0.001). Then, in comparison between the HNCAS group and the healthy control group, VD and SD were significantly lower in the HNCAS group (P_VD_ = 0.02, P_SD_ < 0.001), but FD was comparable between groups (P_FD_ = 0.06). Nevertheless, there were no significant differences in FAZ among the three groups (P_1_ = 0.64, P_2_ = 0.72, P_3_ = 0.36).

**Table 2:** Ocular microcirculation metrics of participants.

Ocular metrics	HCAS *N* = 50 (eyes)	HNCAS *N* = 71 (eyes)	Healthy control *N* = 82 (eyes)	P_1_	P_2_	P_3_
**Retinal microcirculation metrics**						
VD (%)	30.64 ± 4.25	32.62 ± 4.67	34.15 ± 3.16	0.02^*^	<0.001^*^	0.02^*^
SD (%)	13.25 ± 1.92	14.8 ± 2.05	16.7 ± 1.5	<0.001^*^	<0.001^*^	<0.001^*^
FD	1.79 ± 0.04	1.86 ± 0.06	1.88 ± 0.07	<0.001^*^	<0.001^*^	0.06
FAZ (mm^2^)	0.4 ± 0.14	0.39 ± 0.1	0.41 ± 0.16	0.64	0.72	0.36
**Choroidal microcirculation metrics**						
SFCT (μm)	239.11 ± 56.8	249.25 ± 64	242.32 ± 62.4	0.37	0.76	0.5
TCA (mm^2^)	1.80 ± 0.4	1.86 ± 0.36	1.82 ± 0.35	0.15	0.7	0.17
LA (mm^2^)	0.98 ± 0.24	1.13 ± 0.13	1.15 ± 0.23	<0.001^*^	<0.001^*^	0. 3
CVI	54.45 ± 4.63	60.75 ± 9.42	63.2 ± 6.31	<0.001^*^	<0.001^*^	0.06
**Retinal vessel caliber metrics**						
CRVE	0.24 ± 0.03	0.23 ± 0.03	0.23 ± 0.04	0.07	0.13	0.99
CRAE	0.12 ± 0.02	0.13 ± 0.04	0.15 ± 0.03	0.11	<0.001^*^	0.001^*^
AVR	0.5 ± 0.14	0.57 ± 0.29	0.68 ± 0.16	0.12	<0.001^*^	<0.001^*^

CAS, carotid artery stenosis; HCAS, hypertension with CAS; HNCAS, hypertension without CAS; VD, vessel density; SD, skeleton density; FD, fractal dimension; FAZ, foveal avascular zone; SFCT, subfoveal choroidal thickness; TCA, total choroidal area; LA, luminal area; CVI, choroidal vascularity index; CRVE, central retinal vein equivalent; CRAE, central retinal artery equivalent; AVR, artery/vein ratio; P_1_, *p*-value between HCAS group and HNCAS group; P_2_, *p*-value between HCAS group and healthy controls; P_3_, *p*-value between HNCAS group and healthy controls; * statistically significant.

### Choroidal microcirculation metrics

The SFCT ranged from 182 to 315 μm without significant differences among the HCAS group, HNCAS group, and control group (all *p* > 0.05). Meanwhile, the TCA ranged from 1.48 to 2.21 mm^2^ without significant differences among the three groups (all *p* > 0.05). The LA and CVI were also comparable between the HNCAS group and the healthy control group (all *p* > 0.05). However, the LA and CVI were significantly decreased in the HCAS group when compared with the HNCAS group and control group (P_1_ < 0.001, P_2_ < 0.001) (Table 2).

### Retinal vessel caliber metrics

The CRVE, CRAE, and AVR were calculated to evaluate the caliber of retinal central vessels. The corresponding results are summarized in Table 2. There were no significant differences in the CRVE among the three groups (all *p* > 0.05). The CRAE and AVR were also comparable between the HCAS group and the HNCAS group (*p* > 0.05). However, the CRAE and AVR were significantly decreased in the HCAS group and HNCAS group when compared to the control group (all *p* < 0.05).

### Regression analyses

Univariate linear regression analyses and multivariate regression analyses were performed to identify clinical factors significantly associated with retinal VD in the HCAS group and HNCAS group, respectively (Table 3). In the HCAS group, univariate linear regression analysis demonstrated that age, smoking history, IMT, and PSV were significantly associated with retinal VD (all *p* < 0.05). A subsequent multivariate analysis that adjusted for age, gender, smoking history, and hypertension duration demonstrated that carotid metrics including PSV [β = −2.68 (95% CI: −5.66 to −1.28), *p* = 0.02] and IMT [β = −1.08 (95% CI: −2.76 to −0.25), *p* = 0.01] were negatively related to retinal VD. In the HNCAS group, univariate linear regression analysis disclosed that age, hypertension duration, and the MAP were significantly associated with retinal VD (all *p* < 0.05). A subsequent multivariate analysis that adjusted for age, gender, smoking history, and hypertension duration demonstrated that MAP had a negative linear correlation with retinal VD [β = −1.06 (95% CI, −2.78 to −0.04), *p* = 0.02]. However, IMT and PSV were not related to retinal VD in the HNCAS group (all *p* > 0.05).

**Table 3:** Clinical factors associated with retinal vessel density in hypertension with and without CAS patients in linear regression analyses.

	Univariate linear analysis			Multivariate linear analysis		
Factors	*Β*	95%CI	*p*-value	β	95%CI	*p*-value
**HCAS group**						
Age	−0.43	−0.65 to −0.25	0.02^*^	−0.4	−0.72 to −0.33	0.03^*^
Gender (male)	3.16	−0.04 to 5.68	0.38	1.86	−0.01 to 3.6	0.66
Smoking history	−1.46	−2.48 to −0.56	0.01^*^	−0.59	−1.08 to −0.05	0.01
BMI	−6.79	−11.4 to −2.65	0.8			
Hypertension duration	−0.32	−0.8 to 1.92	0.23	−0.22	−0.6 to 1.68	0.63
MAP	1.45	−2.66 to 1.2	0.3			
MOPP	−6.68	−9.88 to 8.53	0.28			
IMT	−1.42	−3.02 to −0.38	0.02^*^	−1.08	−2.76 to −0.25	0.01^*^
PSV	−3.98	−7.98 to −1.62	0.002^*^	−2.68	−5.66 to −1.28	0.002^*^
**HNCAS group**						
Age	−0.21	−0.27 to −0.03	0.01^*^	−0.06	−0.15 to −0.02	0.06
Gender (male)	1.24	−0.21 to 1.68	0.14	2.4	−0.38 to 3.86	0.56
Smoking history	−0.76	−1.26 to 0.58	0.42	−0.35	−0.62 to 0.42	0.4
BMI	−0.68	−0.55 to 0.15	0.65			
Hypertension duration	−0.36	−2.6 to −0.82	0.03^*^	−0.29	−2.4 to 0.55	0.52
MAP	−2.76	−3.35 to −0.21	0.03^*^	−1.06	−2.78 to −0.04	0.02^*^
MOPP	−2.39	−2.6 to 0.35	0.2			
IMT	−2.88	−0.06 to 3.42	0.52			
PSV	6.78	−8.97 to 9.66	0.7			

CAS, carotid artery stenosis; HCAS, hypertension with CAS; HNCAS, hypertension without CAS; BMI, body mass index; MAP, mean arterial pressure; MOPP, mean ocular perfusion pressure; IMT, intima media thickness; PSV, peak systolic velocity; β, unstandardized beta; CI, confidence interval; * statistically significant.

Logistic regression analyses were conducted to explore the factors that were significantly associated with the presence of CAS in hypertension patients (Table 4). The results showed that older age [OR = 2.03 (95% CI: 1.2 to 3.06), *p* < 0.001] and smoking history [OR = 2.61 (95% CI: 2.12 to 4.05), *p* = 0.002] were positively related with CAS in hypertension; however, anti-hypertensive treatment [OR = 0.78 (95% CI: 0.65 to 0.97), *p* = 0.02] was negatively related with CAS in hypertension subjects. Considering ocular microcirculation metrics, VD [OR = 0.48 (95% CI 0.21 to 0.72), *p* = 0.04], SD [OR = 0.35 (95% CI 0.17 to 0.55), *p* = 0.02], FD [OR = 0.79 (95% CI 0.48 to 0.91), *p* = 0.04], and CVI [OR = 0.12 (95% CI 0.03 to 0.42), *p* < 0.001] were negatively related to the CAS. Nevertheless, the retinal vessel caliber metrics, including CRAE, CRVE, and AVR, were independent of CAS in hypertension (all *p* > 0.05).

**Table 4:** Factors associated with CAS in patients with hypertension in logistic regression analysis.

	OR (95%CI)	*p*-value
Age	2.03 (1.2 to 3.06)	<0.001^*^
Gender (male)	1.03 (0.53 to 1.72)	0.5
Gender (female)	1 (Ref)	
BMI	1.32 (0.65 to 2.04)	0.14
Smoking (Y)	2.61 (2.12 to 4.05)	0.002^*^
Smoking (N)	1 (Ref)	
Hypertension duration	1.13 (0.86 to 2.88)	0.4
Anti-hypertensive treatment (Y)	0.78 (0.65 to 0.97)	0.02^*^
Anti-hypertensive treatment (N)	1 (Ref)	
MAP	0.53 (0.22 to 1.06)	0.51
MOPP	0.72 (0.48 to 1.52)	0.08
VD (%)	0.48 (0.21 to 0.72)	0.04^*^
SD (%)	0.35 (0.17 to 0.55)	0.02^*^
FD	0.79 (0.48 to 0.91)	0.04^*^
FAZ (mm^2^)	0.42 (0.09 to 1.02)	0.4
SFCT (μm)	0.88 (0.52 to 1.36)	0.78
CVI	0.12 (0.03 to 0.42)	<0.001^*^
CRVE	2.68 (2.02 to 3.59)	0.45
CRAE	0.92 (0.35 to 1.65)	0.7
AVR	0.86 (0.32 to 1.48)	0.09

CAS, carotid artery stenosis; BMI, body mass index; MAP, mean arterial pressure; MOPP, mean ocular perfusion pressure; VD, vessel density; SD, skeleton density; FD, fractal dimension; FAZ, foveal avascular zone; SFCT, subfoveal choroidal thickness; CVI, choroidal vascularity index; CRVE, central retinal vein equivalent; CRAE, central retinal artery equivalent; AVR, artery/vein ratio; OR, odds ratio; CI, confidence interval; * statistically significant.

## Discussion

This study demonstrated that CAS induced hypoperfusion in retinal and choroidal microcirculation in hypertension patients; moreover, retinal VD and choroidal CVI were associated with the presence of CAS in hypertension patients. The results of this study suggested that the macro- and microvascular complications of hypertension were mutually affected and may share a common pathophysiology. Furthermore, the ocular microcirculation metrics could be used as non-invasive imaging biomarkers for macrovascular diseases which require further monitoring in hypertension.

Our study disclosed significantly decreased VD, SD, and FD in HCAS patients, which was consistent with prior studies (Xu et al., 2022; Yoon et al., 2022). The relationship between carotid blood flow and retinal blood flow has been evaluated by several techniques in the past few years. In the beginning, color Doppler was taken to detect the reduction of blood flow in the ophthalmic artery and posterior ciliary artery in CAS patients and its improvement after surgical treatment (Kawaguchi et al., 2002; Sanjari et al., 2009). Then, fundus photography and fundus fluorescein angiography were considered objective and robust methods for assessing retinal microcirculation in carotid disease (Spertus et al., 1984). Recently, OCTA was developed, and retinal microcirculation metrics, including VD, SD, FD, and FAZ, were extracted from OCTA images to quantify retinal microcirculation. VD is calculated as the percentage area occupied by blood vessels and used as a useful marker for retinal capillary dropout. SD could reflect the real VD in the condition of vessel engorgement because it indicates VD independent of vascular diameter. FD represents the complexity of capillary perfusion, and FAZ reflects the severity of foveal ischemia. Subsequently, there were studies that disclosed decreased retinal VD in CAS patients (Drinkwater et al., 2020; Xu et al., 2022; Yoon et al., 2022) and some pilot studies that detected significant improvement of retinal VD after carotid endarterectomy (Lahme et al., 2018) and carotid angioplasty and stenting (Lee et al., 2019), suggesting the negative effect of CAS on retinal microcirculation. Moreover, the regression analyses confirmed that in HCAS patients, IMT and PSV, which were important predictors for diagnosing and grading CAS, were negatively associated with retinal VD and SD, which further verified that CAS has a negative effect on retinal microcirculation. The logistic regression showed that lower retinal VD and SD were significantly related to the presence of CAS in hypertension patients, which suggested that retinal metrics could be biomarkers for the presence of CAS in hypertension patients. Some studies reported that primary hypertension could decrease retinal VD, reduce the complexity of capillary perfusion, and narrow retinal arterioles (Xu et al., 2021; Hein et al., 2023; Kocer et al., 2023). Similarly, we also detected lower VD, SD, and FD in the HNCAS group when compared with healthy control, which disclosed that primary hypertension could decrease retinal blood perfusion. The negative relationship between MAP and retinal VD in regression analysis further verified the negative effect of hypertension on retinal microcirculation. As we know, hypertension is one of the independent risk factors for CAS, and CAS is one of the most common macrovascular complications of hypertension (Drinkwater et al., 2020). However, there was no study that researched the relationship between CAS and retinal microcirculation in hypertension. This study first showed that in hypertension patients, CAS induced lower retinal VD and SD. Meanwhile, the lower retinal VD and SD were biomarkers for the presence of CAS. The pathogenic mechanism of how CAS and retinal microcirculation interact with each other is not well-established, although there are several hypotheses. One theory believes that micro- and macro-complications of hypertension may share similar risk factors, such as age, gender, and smoking, which could contribute to both diseases. In addition, another theory believed that hypertension macroangiopathy evolves from microvascular damage because recent evidence has shown that the vasa vasorum of the macro-vessels in diabetic patients shared a similar process as the microvascular complication: First, endothelial cell dysfunction and vascular permeability increased, then hypoxia may result in angiogenesis and neovascularization (Rubinat et al., 2015). Hence, we believe that CAS and retinal microcirculation in hypertension are mutually Affected and interconnected. Non-invasive retinal microcirculation metrics assessed by OCTA could be used as early indicators of macrovascular complications of hypertension, which requires further monitoring and screening.

We detected lower LA and CVI in the HCAS subjects. To the best of our knowledge, this is the first study that investigated the effect of CAS on choroidal microcirculation and explored the relationship between carotid metrics and choroidal metrics in hypertension patients. A total of 70% of blood flow from the ophthalmic artery goes to the choroid (Mrejen and Spaide, 2013). Hence, we hypothesized that the hypoperfusion state of CAS may cause a change in choroidal microcirculation. Our finding was consistence with the study reported by Wang J et al., who utilized SS-OCTA to identify a decrease in choroidal blood flow among CAS patients, and the choroidal blood flow inversely correlated with the severity of CAS (Wang et al., 2019). We speculated that CAS could induce choroidal hypoperfusion, and then choroidal hypoperfusion may cause choriocapillaris occlusion, choroidal vessel attenuation, choroidal ischemia, and infarct, leading to the lower LA and CVI in the HCAS group. The CVI is an effective indicator of choroidal blood perfusion. Inflammation leads to hyperemia in the choroid, resulting in a higher CVI, while hypoperfusion causes ischemia in the choroid, leading to a lower CVI. The regression analyses further verified that lower CVI was significantly related to the presence of CAS in hypertension patients. Prior studies showed that SFCT thinned significantly in CAS patients and thickened after carotid endarterectomy (Lareyre et al., 2018; Ala-Kauhaluoma et al., 2021). In this study, there was no significant difference in SFCT among HCAS patients, HNCAS patients, or healthy controls. The contrary conclusions of the SFCT could be explained by two reasons: 1. SFCT was determined using Haller’s layer, the absence of change in SFCT may suggest that the large vessels in Haller’s layer are unable to react to the hypoperfusion state of CAS or the hypertensive state of hypertension; 2. A prior study reported that in the early stages of CAS, choroidal collateral vessels dilate to compensate for reduced blood supply, which may cause choroidal thickening (Yeung et al., 2020). Then, in the middle or late stage of CAS, the hypoperfusion state of CAS will cause occlusion in choriocapillaris, which may cause choroidal thinning. We included patients with CAS without considering the severity of CAS as a confounding factor, which may lead to misleading results and necessitate further investigation in future studies. In conclusion, we hypothesize that CAS has a detrimental impact on choroidal microcirculation in hypertensive patients due to reduced choroidal perfusion caused by CAS. Furthermore, the CVI could serve as a valuable biomarker for detecting the presence of CAS in individuals with hypertension. Additionally, no significant differences were observed in choroidal metrics between hypertensive patients with and without CAS, suggesting that hypertension alone does not affect choroidal microcirculation. However, previous studies have reported choriocapillaris flow deficits using SS-OCTA in chronic hypertension (Chua et al., 2021), and thinner SFCT has been documented in hypertensive patients by Güven et al. (2023). These contradictory findings may be attributed to the oversight of considering the effects of CAS on the choroid in prior investigations. The influence of chronic hypertension on choroidal microcirculation remains unclear and warrants further research.

Our study disclosed that there were no significant differences in CRVE and CRAE between HCAS and HNCAS groups, which suggested that CAS has no effect on retinal vessel caliber in hypertension subjects. Blood flow was determined by vessel caliber; even a small change in vessel caliber could result in a large change in blood flow. Measuring vessel diameter directly from a fundus photograph was inaccurate and one-sided. Hence, CRAE and CRVE were summarized to quantify vessel caliber and blood flow. Our result was consistent with that of Xu et al. They reported that there were no significant differences in retinal artery/vein calibers between CAS and control (Xu et al., 2022). However, Marianne et al. reported that CRVE was higher in CAS patients and decreased after surgery. CRAE was comparable between CAS and control and unchanged after surgery (Ala-Kauhaluoma et al., 2023). Meanwhile, Machalinska et al. found decreased CRAE in 65 asymptomatic CAS patients (Machalińska et al., 2019). The contrary conclusions on the relationship between CAS and retinal vessel caliber should be due to ignoring the effect of BP on retinal vessel diameter in prior studies. As we know, most CAS patients have hypertension, and hypertension could cause arteriolar narrow and venular tortuosity because of increased peripheral vascular resistance. Prior studies detected significant changes in retinal vessel caliber in CAS patients without considering hypertension as a confounding factor; hence, the change in retinal vessel caliber in CAS patients may be attributed to hypertension other than CAS. In our study, CRAE and AVR were lower in HNCAS than in healthy control, which confirmed the effect of hypertension on retinal vessel caliber.

Several limitations should be acknowledged in this study. First, this cross-sectional study included a limited number of subjects. Second, ocular factors such as axial length, refractive degree, and IOP were not considered potential confounders in retinal and choroidal circulation. Third, due to unresolved artifacts from SRL on the deep retinal layer at 6 × 6 mm scans, the microcirculation in the deep retinal layer was not investigated in this study. Fourth, it is important to note that CVI served as a quantified index of the choroidal vascular component rather than representing the true VD of the choroid. Additionally, only the ipsilateral eye of CAS patients was included without considering the contralateral eye. Therefore, further research is needed to explore both eyes comprehensively.

In conclusion, we found that CAS had negative effects on retinal and choroidal microcirculation; meanwhile, lower retinal VD and choroidal CVI were risk factors for the presence of CAS in hypertension patients, which implied macro- and microangiopathy in hypertension were mutually affected and may share the common pathophysiology. Moreover, non-invasive assessment of ocular microcirculation metrics could potentially serve as indicators for evaluating CAS risk profile among hypertensive individuals.

## Data availability statement

The original contributions presented in the study are included in the article/supplementary material, further inquiries can be directed to the corresponding author.

## Ethics statement

The studies involving humans were approved by Ethics Committee of Affiliated hospital of Qinghai University. The studies were conducted in accordance with the local legislation and institutional requirements. Written informed consent for participation was not required from the participants or the participants’ legal guardians/next of kin in accordance with the national legislation and institutional requirements.

## Author contributions

JM: Conceptualization, Data curation, Formal analysis, Funding acquisition, Methodology, Supervision, Writing – original draft, Writing – review & editing. NG: Conceptualization, Data curation, Investigation, Resources, Writing – review & editing. MZ: Data curation, Methodology, Software, Writing – review & editing. XM: Investigation, Software, Writing – review & editing. CH: Investigation, Software, Writing – review & editing.
